# Effect of Soil Washing with an Amino-Acid-Derived Ionic Liquid on the Properties of Cd-Contaminated Paddy Soil

**DOI:** 10.3390/toxics11030288

**Published:** 2023-03-20

**Authors:** Yun Deng, Sheng Wang, Ian Beadham, Xin Gao, Mengmeng Ji, Guang Wang, Changbo Zhang, Wenquan Ruan

**Affiliations:** 1 School of Environment and Civil Engineering, Jiangnan University, Wuxi 214122, China; 2 School of Pharmacy and Chemistry, Kingston University, Kingston upon Thames KT1 2EE, UK; 3 National Key Laboratory of Water Environment Simulation and Pollution Control, South China Institute of Environment Sciences, Ministry of Ecology and Environment of the People’s Republic of China, Guangzhou 510665, China; 4 Agro-Environmental Protection Institute, Ministry of Agriculture and Rural Affairs, Tianjin 300191, China

**Keywords:** amino-acid-derived ionic liquids, Cd-contaminated soil, wash, soil properties

## Abstract

To reduce contamination levels in Cd-contaminated paddy soil while retaining soil characteristics, we have studied the Cd-removing ability of 15 different amino acid-based ionic liquids, which are considered to be green solvents, as soil washing agents and their impact on soil. The results indicated that the glycine hydrochloride ([Gly][Cl]) removed the most Cd, and under optimized conditions could remove 82.2% of the total Cd. Encouragingly, the morphology of the soil had not been significantly changed by the washing process. After the soil was rinsed twice with water and the pH was adjusted to 6.2 by adding Ca(OH)_2_, the germination index of the rice increased by 7.5%. The growth of the rice was also stimulated, with lengths and weights of the rice plants increasing by 56% and 32%, respectively, after two weeks. These experiments demonstrate that amino-acid-derived ionic liquids can be promising soil-washing agents of Cd-contaminated paddy soil.

## 1. Introduction

Today, anthropogenic industrial and agricultural activities expose the environment to massive volumes of pollutants, especially in developing countries [1]. In China, 2 × 10^7^ hectares of farmland are polluted by heavy metals, which is caused by sewage irrigation, sludge application, mining, and smelting operations for metallic ores [2]. 56–59% of dietary cadmium exposure comes from rice.The Chinese Standard for Soil Pollution Risk Control of Agricultural Land (GB 15618-1996) has set a critical guideline value of 0.40 mg L^−1^ for agricultural soil with a pH value of 5.5–6.5, and 0.30 mg L^−1^ for soil with a pH value lower than 5.5. However, the technologies available for farmland soil remediation are very limited. Conventional techniques, such as soil replacement, soil isolation, vitrification, encapsulation, and/or soil washing, would affect crop production. Hence, in situ chemical stabilization and agronomic management are the most applied methods at present, both of which hardly reduce the total amount of Cd in soil and do not solve the long-term risks associated with Cd-contaminated soil.

In contrast, phytoextraction and soil washing permanently remove metals from soils. Soil washing using alkaline solvents, organic and inorganic acids, phosphates, surfactants, and chelators has demonstrated remarkable efficiency in reducing heavy metal-contaminated soil [3,4,5,6]. Furthermore, its simplicity and high speed of operation present soil washing as a potential approach for the remediation of heavy-metal-contaminated soil. During the last two decades, ethylenediaminetetraacetic acid (EDTA) has attracted plenty of attention due to its high efficiency and thermodynamic stability of the formed metal complexes [7]. Nevertheless, soil washing also has some limitations. For example, its efficiency is poor for soil with a high clay content, due to its poor permeability [8]. Therefore, this technique usually employs physical processes to separate the polluted soil particles prior to washing.

Another major obstacle to the widespread acceptance of soil washing as a strategy for environmental remediation is that potential ecological risks and loss of soil function seem to be insurmountable mountains for its practical application [9]. For example, inorganic acids (e.g., HCl and H_3_PO_4_) [10] damage the soil, while treatment with salts (e.g., CaCl_2_, FeCl_3_) can lead to nutrient loss and decreased soil fertility [11]. Synthetic chelating agents (e.g., EDTA) [12] and surfactants (e.g., Triton X-100) [13] are often resistant to biodegradation, are toxic, or cause secondary pollution, thus contributing to potential adverse effects on soil functioning [14]. As a consequence, more environmentally friendly washing agents are being investigated for the purpose of promoting the application of soil washing, such as natural amino acids [15], dissolved organic matter [16,17,18], magnetic (magnetite or maghemite) nanoparticles functionalized with chelating agents [19], poly-glutamic acid [20], and saponins combined with deep eutectic solvents [21]. In addition, the washing process would produce a lot of stable metal complexes during wastewater loading, which are hard to treat and increase the cost [22,23].

Ionic liquids (ILs), a type of organic salt with melting points lower than 100 °C, have become known as green substitutes for organic solvents over the past decade. Since their properties can be adjusted by their structural design according to people’s needs, they are considered to be fascinating “designable” chemicals, “future solvents” [24], and “panacea” solvents [25] that can solve many bottleneck problems across various domains. ILs show high extraction capacity for plenty of metal ions, such as nickel [26], palladium [27], and lead [28]. However, ILs are rarely investigated for use in soil remediation because the commonly used ILs, imidazolium- and pyridinium-based ILs, are not as green as desired. This is because the imidazolium and pyridinium cations are resistant to biodegradation and somehow toxic [29]. For the purpose of developing greener ILs, Tao et al. [30] synthesized a series of ILs using amino acids as the cationic precursor by easily mixing an amino acid (weak base) and a relatively strong acid in a suitable molar ratio. These amino-acid-derived ILs (AA-ILs) are prepared using bio-renewable natural compounds as starting materials in a one-step procedure, which is a typical atom-economic reaction without any poisonous by-products. Since the structure of amino acid is retained in the cation of the IL, the cation has similar biodegradability to its precursor amino acid, whilst the toxicity and resistance to biodegradation of imidazolium-based ILs are normally caused by the imidazole cation [31]. This generation of “fully green” ILs proposed the possibility of using ILs in areas where “green” chemicals are needed. 

In this study, the potential for AA-ILs as washing agents was assessed for the Cd-contaminated paddy soil, including the effectiveness of Cd removal and the impact on the soil. The objectives of the work were to: (1) choose an AA-IL from 15 AA-ILs which can remove Cd from soil effectively, and verify if Cd in the wastewater can be removed easily; (2) verify whether the washing destroys the function of the paddy soil; and (3) discuss the potential mechanisms of Cd removal using AA-IL. This work could provide a possible green and sustainable solution for remediation of heavy-metal-contaminated farmland. 

## 2. Materials and Methods

### 2.1. Soil and Reagents

Soil samples were collected at 6 sites at a depth of 0–20 cm from a paddy field in Xiangtan, Hunan Province, China. After collection, all the samples were mixed evenly and then aged for 3 months at room temperature. Then, the soil was air-dried at 25 ± 2 °C for 30 days and ground to pass through a 2 mm sieve after crushing. The concentration of Cd in the soil was 1.31 ± 0.06 mg kg^−1^. According to GB 15618-1996, the content of Cd in the soil exceeds the critical guideline value of 0.40 mg kg^−1^. The other basic properties of the soil are shown in Table 1.

The amino acids and inorganic acids were purchased from Sinopharm Chemical Reagent Co., Ltd. (Shanghai, China). The standard reference Cd solution (1000 mg/L in 1 mol/L nitric acid solution, No. GSB 04-1721-2004) was purchased from National Nonferrous Metals and Electronic Materials Analysis and Test Center (Beijing, China). The water used in this study was deionized, and came from an ultra-pure water machine (EPED-20^TH^, Shanghai, China).

**Figure 1:**
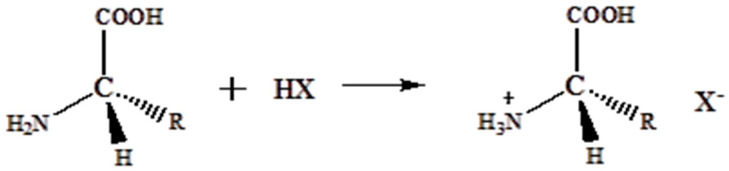
Synthesis of [AA][X]. AA = Gly (glycine), Phe (phenylalanine), Thr (threonine), Glu (glutamic acid), and Lys (lysine). X = Cl^−^, NO_3_^−^, 1/3 PO_4_^3−^.

The ILs [AA][X] were synthesized by following the reported protocol [24]. The amino acid and an inorganic acid were mixed in an equimolar ratio (or 3:1 for H_3_PO_4_) in aqueous solution. The solution was then agitated with a magnetic stirrer for 8 h at 60 °C. After the reaction (Figure 1) was complete, water was removed using a rotary evaporator. The resulting [AA][X] salts were obtained either as white powders or yellow oils. The solutions of each IL at a concentration of 0.3 mol/L (pH values in Table 2) were prepared as washing solutions. 

### 2.2. Cd Removal Percentage of Soil Washing

A solution of an IL at a concentration of 0.3 mol/L was mixed with the soil in a ratio of 4:1 (*r_solution/soil_*) (*w*/*w*). The mixture was then agitated in an orbital shaker at 150 r/min for 6 h at 25 ± 1 °C, and centrifuged at 3000 rpm for 10 min. Cadmium concentration in the supernatant (C_Cd_) was determined by a flame atom absorption spectrometer (AA-7000, Shimadzu, Kyoto, Japan). The standard curves were prepared using a standard reference Cd solution. To avoid the impact of solid–liquid separation on the removal effect, we assumed complete separation of solution and soil, and the calculated percentage Cd removal (*E*%) was determined using Equation (1):

E_Cd_% = (V × C_Cd_)/(m × C_0_) (1)

where V is the volume of [AA][X] solution (mL); m is the mass of the soil (g); and C_0_ is the Cd(II) concentration in the soil before washing (mg kg^−1^).

The concentrations of different Cd fractions in the soil before and after washing were tested using a method modified from Tessier et al. [32]. Table 3 presents the detailed steps of the Tessier continuous extraction method. The five Cd fractions are: exchangeable Cd (EXCH), the carbonate fraction (CARB), Cd bound to Fe and Mn oxides (FeMnOx), Cd bound to organic matter (OM), and residual Cd (RESI). The degrees of compliance of the balance of Cd were 95.7–104.6%.

### 2.3. Analysis of Soil Properties

The soil was characterized by an X-ray diffractometer (D2 PHASER, Bruker, Karlsruhe, Germany) and a Fourier-transform infrared spectrometer (Tracer-100, Shimadzu, Kyoto, Japan). The major elements in the soil were analyzed using a scanning electron microscope equipped with an X-ray energy dispersion spectrometer (SEM-EDS) (S-4800, Hitachi, Tokyo, Japan) at an accelerating voltage of 40 kV and a beam current of 100 μA. The pH of the soil was determined in the supernatant from the soil–water mixture, 1:2.5 (*w*/*w*), using a pH meter (SC-619, Mettler Toledo, Zurich, Switzerland). The total nitrogen (TN), total phosphorus (TP), total potassium (TK), available potassium (AK), and available phosphorus (AP) were also determined according to standard methods [33]. 

### 2.4. Rice Cultivation in the Soil

The soil pH was adjusted to 6.2 ± 0.2 by treatment with 1% Ca(OH)_2_ solution. Then, 100 g of the drained soil, 80 g of deionized water, and 40 rice seeds were incubated in a Petri dish at 27 °C for a week. The germination index (GI) of the rice was calculated using the following equation:

GI = G^S^/G^0^ × 100% (2)

where G^S^ and G^0^ are the number of germinated seeds in the sample and the control, respectively. After incubation for two weeks, ten rice seedlings were taken from each Petri dish. After cutting the roots, the lengths of the seedlings were measured. Then, the seedlings were washed and dried in an oven at 105 °C for 1 h, then at 50 °C until the weight remained constant. The weights of the seedlings were measured, and the reported data represent averages of ten seedlings.

As all experiments were performed as replicates, the results are expressed as the average value ± standard error. Matrix-spiked parallel samples and method blank samples were also prepared and analyzed as quality control, and the test results show that the relative deviation of all parallel samples is within the allowable relative deviation range. Statistical analysis of data was performed using the IBM SPSS Statistics 20.0 and the results with a significant difference are at a level of *p <* 0.05.

## 3. Results

### 3.1. Effectiveness of Cd Removal and Wastewater Treatment

**Figure 2:**
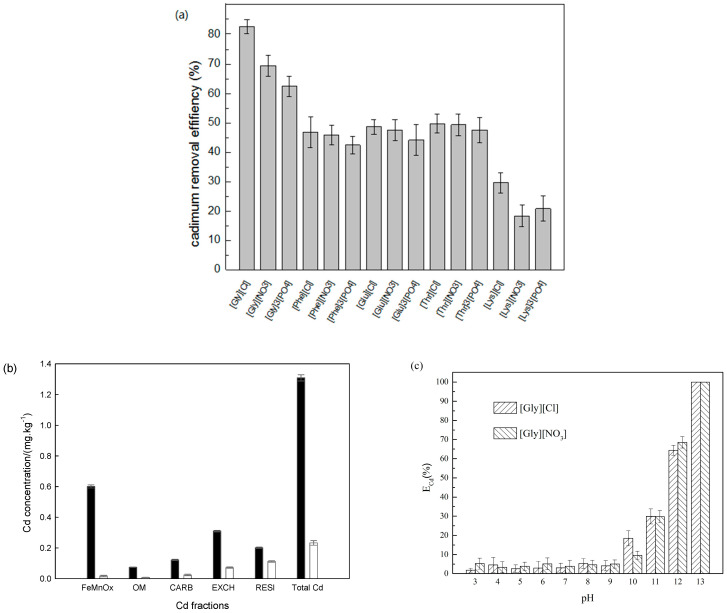
(**a**) Percentage Cd removal by different ILs; (**b**) distribution of Cd fractions in the soil before and after washing with [Gly][Cl]. The black and white bars represent soil before and after washing, respectively; (**c**) Cd removal percentage in washing wastewater with [Gly][Cl] at different pHs.

Assuming complete separation of solution and soil, the calculated percentages of Cd removal (*E*%) for the 15 ionic liquids ranged from 18.3% to 82.2%. In the control experiments using an aqueous solution of HCl (pH 1.5, the same pH with [Gly][Cl] solution) and glycine (0.3 M), the Cd removal percentage (*E*%) was 38.2% and below the detection limit, respectively. Using [Gly][Cl], *E*% was 82.2% and Cd concentration in the washed soil was 0.23 mg kg^−1^, lower than 0.4 mg kg^−1^—the risk screening value in GB 15618-2018. After washing with [Gly][Cl], the vast majority of FeMnOx was removed, followed by OM, CARB, and EXCH (Figure 2b). Less than half of the residual fraction (RESI) was removed by washing, making it the most abundant fraction after washing. 

[Gly][X] had the highest *E*% (62.5–74.5%), possibly because [Gly]^+^ has excellent solubility and exhibits little steric hindrance, as [Gly]^+^ is the smallest cation. For [Phe][X], [Thr][X], and [Glu][X], more moderate *E*%s were recorded, ranging from 42.5% to 49.8%. Although [Glu]^+^ has two carboxyl groups and [Thr]^+^ has an additional hydroxyl, which are usually regarded as ligating groups, their salts still exhibited lower *E*% values than [Gly][Cl]. [Lys][X] had the lowest *E*% (18.3–29.6%), despite [Lys]^+^ having a similar molecular weight to [Glu]^+^, and a ligating side-chain amino group. These results suggest that side-chain oxygen- and nitrogen-donor ligands may in fact be detrimental to Cd extraction. This may be a result of enhanced amino acid complexation to alternative metal acceptor sites on the soil particles, rather than Cd, if side-chain ligands are present.

For salts with the same cation, Cl^−^ AA-ILs generally reduced more Cd than either the PO_4_^3−^ or NO_3_^−^ salts. This may be due to the lower pH of Cl^-^AAILs, because hydrochloric acid is a stronger acid than either phosphoric or nitric acid. More rapid Cd dissociation of the AA-Cd chloride complex from the soil may also be involved, as Cd^2+^ desorption from soil is more favorable in the presence of Cl^−^ compared to NO_3_^−^ [34]. The equilibrium, Cd^2+^ + yCl^−^ ⇄ CdCl_y_^2−y^, is favored in the presence of Cl^−^ [10,11] and the formation of stable Cd–Cl complexes disfavors re-adsorption of Cd^2+^ onto adsorption sites on the surface of soil particles [35]. PO_4_^3−^ also forms complexes with Cd^2+^, but the resulting Cd-PO_4_ complexes are significantly less water-soluble [36]. Correspondingly, PO_4_^3−^-AAILs exhibited the lowest Cd capacity for Cd removal. 

Wastewater treatment was straightforward, requiring only the addition of aqueous NaOH. When the resulting alkaline wastewater had reached pH 10, Cd began to precipitate, and at pH 13, Cd was no longer detectable in the solution (Figure 2c).

### 3.2. Impact on Soil Properties

**Figure 3:**
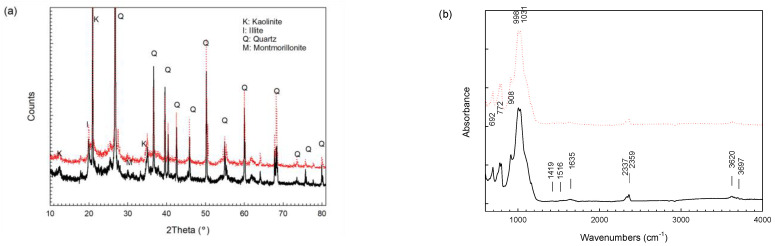
(**a**) XRD and (**b**) FTIR spectra of soil before and after washing. The solid black line represents soil before washing and the dotted red line represents soil after washing.

The XRD and FTIR spectra (Figure 3) indicated that the soil consisted of both clay minerals (viz., kaolinite, illite, and montmorillonite) and non-clay minerals (viz., quartz). After washing, the mineral composition of the soil did not obviously change. The presence of bands at 1419 cm^−1^ and 1516 cm^−1^ in the FTIR spectra of the soil after washing is probably attributed to methylene and secondary amide of glycine, respectively.

**Figure 4:**
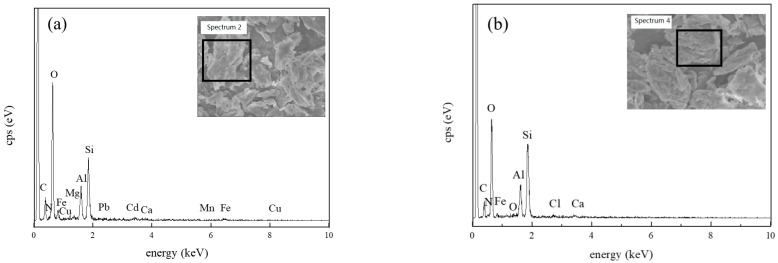
SEM-EDS analysis of soil (**a**) before washing; (**b**) after washing with [Gly][Cl].

The morphology and major elemental composition of the soils after washing were analyzed by SEM-EDS. The morphology of the soil had not changed significantly (Figure 4). The percentages of C, N, O, and Cl had increased because of residual glycine salts (Table 4). Additionally, Mn was no longer detected and the levels of Fe had decreased by over 45%. Because of the high affinity of heavy metals for soil constituents, including silicates, metal oxides, and organic matter, effective metal dissolution is an essential prerequisite for the complete removal of heavy metals [37]. The metal-solubilizing effect of the glycine salts was evident when concentrations of Al and Mg had also decreased, indicating that Al, Mg, and Fe oxides possibly had partially dissolved during remediation. Cd, Cu, and Pb were no longer detectable after washing with [Gly][Cl], indicating that [Gly][Cl] can possibly remove the four heavy metals of Cd, Mn, Cu, and Pb at the same time.

The nutrient content has been determined (Table 4). Percentages of organic matter (Or), nitrogen (N), and available phosphorus (AP) in the soil were elevated, while the potassium (K) and total phosphorus (TP) content declined after washing. The increase in AP, accompanied by a decline in total phosphorus (TP), may either be explained by the dissolution of phosphorus bound to secondary minerals or by decreased phosphorus sorption by organominerals, due to ligand exchange and ligation of phosphorus by Fe and Al [38], as has been reported for low-molecular-weight organic acids. 

**Figure 5:**
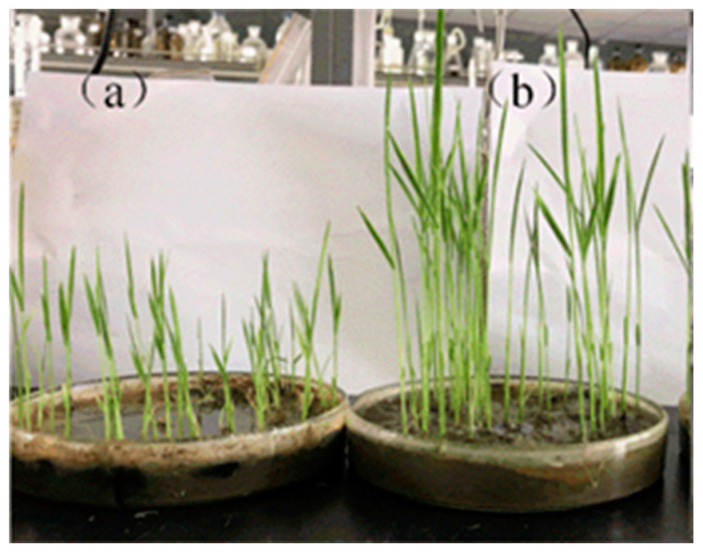
Rice cultivated: (**a**) in original soil; (**b**) in soil washed with [Gly][Cl].

After rinsing twice with water and adjusting the soil pH to 6.2 ± 0.2 by addition of Ca(OH)_2_, rice plants were grown in the remediated soil. The germination index (GI) of the rice had increased to 87.5%, which was 7.5% higher than the GI for the rice planted in the original soil, prior to remediation (Table 4). Growth of the rice had also been promoted (Figure 5), with plant lengths increased by 56% and weights by 32% after washing (Table 4). This promotion of rice growth is likely to be a result of heavy metal remediation, combined with the effect of added organic carbon and nitrogen from the IL, and higher calcium levels from Ca(OH)_2_ treatment. 

### 3.3. Potential Cd Removal Mechanism

[Gly][Cl] is a salt formed from a strong acid and weak alkali, and its aqueous solution is modestly acidic (the pH of a 0.3 M solution is 1.5). Correspondingly, the extraction of Cd from soil may involve similar mechanisms to those observed when using other acids, salts, ligands, and chelating agents. 

The basic soil components, such as Fe–Mn oxides, aluminum oxides, and metal carbonates, may be partially dissolved [10,11]. Furthermore, the H^+^ and amino acid cations may exchange with Cd(II) on the reactive surface sites of the soil matrix [11,39]. Protons can also react with soil surface sites (layer silicate minerals and/or surface functional groups, e.g., Al-OH, Fe-OH, and CO_2_^−^ groups) and enhance desorption of Cd(II) cations [40]. In addition, the change in pH also destabilizes adsorbed Cd by favoring both the soil-Cd hydrolysis equilibrium and co-precipitation of Cd in soil.

In addition, the amino and carboxyl groups of the glycine [9], as well as Cl^−^, may coordinate with cadmium to form stable complexes, which are not re-adsorbed onto soil surfaces. The mode of complexations between Cd and the IL could be accompanied by ligation of the amino and/or carboxyl to the metal. Other IL-soluble Cd(II) complexes (Cd([Gly][Cl])_n_Cl_y−n_) are plausible, and their formation can be summarized by Equation (3):

(3) $${\left[{\mathrm{CdCl}}_{y}\right]}^{2-y}+n\left[\mathrm{Gly}\right]\left[\mathrm{Cl}\right]\to \mathrm{Cd}{\left(\left[\mathrm{Gly}\right]\left[\mathrm{Cl}\right]\right)}_{n}{\mathrm{Cl}}_{y-n}+n\mathrm{HCl}$$

Therefore, the formation of Cd complexes may either prevent cadmium from being adsorbed by soil again, or it may transfer the Cd into the aqueous phase [39,41].

## 4. Discussion

With [Gly][Cl], most Cd can be removed. The labile species, EXCH and CARB, were less effectively extracted than OM, probably because of Cd re-adsorption from other fractions into the exchangeable fraction as a result of polar interactions [42]. Other studies, e.g., the reduction of Pb using EDTA and EDDS (strongly Fe^3+^/Mn^2+^ chelating ligands), have shown differing results from Wei’s findings, with EXCH and CARB fractions being more effectively extracted than the FeMnOx fraction [43]. These results indicated that differences in metal affinity towards the extractant are of critical importance. Unexpectedly, in another study into EDTA extraction by Liang et al. [42], the EXCH and CARB fractions were far less effectively extracted than the FeMnOx fraction. Liang’s findings suggested that factors other than the nature of the ligand are involved, and in an important publication, Sun et al. found considerable variation between the five fractions when studying four different soils. Sun et al.’s thorough study confirmed that Cd extractability was not only determined by fraction lability in the presence of a ligand, but also by the kinetics of metal desorption/dissolution and the mode of washing agent addition for a particular soil composition [44]. Therefore, the removal efficiency of AA-ILs for heavy metals in soil may also change with different leaching methods and soil properties. 

The metal oxides present in soil have possibly been partially dissolved, resulting in the possible loss of Fe, Mg, and Mn. The loss of soil mineral elements, such as Ca, Mg, Fe, and Al, is usually observed in the washed soil with other agents [45]. Washing with EDTA may cause more than 50% loss of Ca [46]. The oxides are important constituents of the soil, as they regulate the absorption and desorption of both nutrients and potentially toxic elements in the soil [47]. Despite less Ca and Al loss in our study, the chlorine ions that remained in the soil may also negatively impact the plants [48]. Therefore, although the growth of rice is promoted in this study, we should also pay attention to the long-term impact. 

After addition of Ca(OH)_2_, the growth of rice in the washed soil was improved. It is an encouraging result because the soils that were washed with many of the other agents exhibited ecotoxicological effects. The seed germination rates decreased dramatically by 3.6–32.1% after soil washing with HCl, H_3_PO_4_ [49], EDTA, FeCl_3_, and mixed chelators [50]. The possible reasons for this include the increase in exchangeable heavy metal content, the change in pH, the toxicity of residual washing agent, and the loss of nutrition [49,50]. In this study, N, AP, and Or were increased, whilst the other toxic elements, such as Pb, Cu, and Mn, were possibly reduced by washing. 

## 5. Conclusions

To develop green washing agents for heavy-metal-polluted soil, this work proposed AA-IL [Gly][Cl]. It showed an effective percentage of Cd removal, 82.2%, from paddy soil. After the final pH adjustment, the germination and growth of rice were improved after washing with [Gly][Cl]. The results proved that the function of the paddy soil was not damaged by washing, and in some cases, was improved. Wastewater from the process was easily treated by simply increasing the pH to precipitate Cd(OH)_2_. From these results, it is clear that amino acid salts show great promise as economical and effective soil-washing agents.

## Figures and Tables

**Table 1:** Characteristics of tested soils.

pH	OM (%)	Particle Size Distribution		
		Sand (%)	Silt (%)	Clay (%)
6.20 ± 0.26	12.42 ± 0.12	29.60 ± 0.14	55.04 ± 0.43	16.36 ± 0.21

Note: OM (organic matter), sand (2–0.22 mm), silt (0.02–0.002 mm), clay (<0.002 mm).

**Table 2:** pH values for 0.3 mol/L aqueous solutions of the AA-ILs.

Salt	pH	Salt	pH	Salt	pH	Salt	pH
[Phe][Cl]	1.49	[Gly][Cl]	1.51	[Glu][Cl]	1.47	[Lys][Cl]	1.66
[Phe][NO_3_]	1.60	[Gly][NO_3_]	1.62	[Glu][NO_3_]	1.53	[Lys][NO_3_]	1.72
[Phe]_3_[PO_4_]	1.61	[Gly]_3_[PO_4_]	1.65	[Glu]_3_[PO_4_]	1.55	[Lys]_3_[PO_4_]	1.73

**Table 3:** Detailed steps of the Tessier continuous extraction method.

Fraction	Reagents	Methods
EXCH	8 mL 1 mol L^−1^ MgCl_2_ (pH = 7.0)	1 h shaking at room temperature
CARB	8 mL 1 mol L^−1^ CH_3_COONa (adjusted to pH = 5.0 with CH_3_COOH)	5 h shaking at room temperature
FeMnOx	20 mL 0.04 mol L^−1^ NH_2_OH·HCl in 25% (*v*/*v*) CH_3_COOH	3 h shaking at 96 ± 3 °C
OM	3 mL of 0.02 mol L^−1^ HNO_3_ and 5 mL 30% H_2_O_2_ (adjusted to pH = 2 with HNO_3_)	2 h intermittent shaking at 85 ± 2 °C
	3 mL 30% H_2_O_2_ (adjusted to pH = 2 with HNO_3_)	3 h intermittent shaking at 85 ± 2 °C
	5 mL 3.2 mol L^−1^ CH_3_COONH_4_ in 5% (*v*/*v*) HNO_3_	0.5 h shaking at room temperature
RESI	HNO_3_:HCl:HF = 6:3:2	25 min microwave digesting at 185 °C

**Table 4:** Properties of the soil (**a**) before washing; (**b**) after washing with [Gly][Cl].

Properties	Parameter	a	b
Major elements content (wt%) by SEM-EDS	C (Cd)	0.13	n.d.
	C (O)	44	47.17
	C (C)	15.2	20.9
	C (N)	1.2	1.98
	C (Cl)	n.d.	0.74
	C (Si)	16.8	16.4
	C (Fe)	11.3	4.86
	C (Mn)	0.19	n.d.
	C (Al)	8.84	7.25
	C (Mg)	0.57	0.33
	C (Ca)	0.39	0.35
	C (Cu)	0.35	n.d.
	C (Pb)	1	n.d.
Nutrient content (g/kg)	C (Or)	13.52 ± 0.21	16.34 ± 0.25
	C (N)	1.29 ± 0.18	1.66 ± 0.23
	C (TP)	0.62 ± 0.03	0.45 ± 0.02
	C (AP)	0.052 ± 0.002	0.115 ± 0.003
	C (K)	17.55 ± 0.25	14.97 ± 0.30
	pH	6.20 ± 0.26	2.71 ± 0.36
Growth metrics for rice seedlings cultured	Length (cm)	7.86 ± 1.21	12.26 ± 1.02
	Weight (g)	0.056 ± 0.005	0.074 ± 0.007
	GI (%)	80.0 ± 0.4	87.5 ± 0.6

## Data Availability

Not applicable.
